# The ethology of empathy: a taxonomy of real-world targets of need and their effect on observers

**DOI:** 10.3389/fnhum.2013.00488

**Published:** 2013-08-22

**Authors:** Stephanie D. Preston, Alicia J. Hofelich, R. Brent Stansfield

**Affiliations:** ^1^Ecological Neuroscience Laboratory, Department of Psychology, University of MichiganAnn Arbor, MI, USA; ^2^Department of Medical Education, University of MichiganAnn Arbor, MI, USA

**Keywords:** empathy, altruism, perception-action, prosocial, sympathy, compassion, helping

## Abstract

Empathy is inherently interpersonal, but the majority of research has only examined observers. Targets of need have been largely held constant through hypothetical and fictionalized depictions of sympathetic distress and need. In the real world, people's response to life stressors varies widely—from stoicism to resilience to complete breakdown—variations that should profoundly influence the prosocial exchange. The current study examined naturally-varying affect in real hospital patients with serious chronic or terminal illness during videotaped interviews about quality of life. Participants viewed each video while psychophysiological data were recorded and then rated each patient's and their own emotion. Patients displayed three major emotion factors (disturbed, softhearted, and amused) that were used to classify them into five basic types (*distraught, resilient, sanguine, reticent, wistful*). These types elicited four major emotions in observers [personal distress (PD), empathic concern (EC), horror, pleasure], two of which were never discovered previously with fictionalized targets. Across studies and measures, *distraught* targets usually received the greatest aid, but approximately as many observers preferred the positive and likeable *resilient* patients or the quietly sad *wistful* targets, with multiple observers even giving their greatest aid to *sanguine* or *reticent* targets who did not display distress or need. Trait empathy motivated aid toward more emotive targets while perspective taking (PT) motivated aid for those who did not overtly display distress. A second study replicated key results without even providing the content of patients' speech. Through an ecological examination of real need we discovered variation and commonality in the emotional response to need that interacts strongly with the preferences of observers. Social interactions need to be studied in ethological contexts that retain the complex interplay between senders and receivers.

The prosocial response has been studied in social, personality, and developmental psychology for decades, revealing largely consistent findings across researchers and populations (reviewed in Eisenberg and Miller, 1987; Preston and de Waal, 2002; Batson, 2011). In order to reliably elicit prosocial responses in the laboratory, virtually all studies used sympathetic, fictional, single targets of need depicted through written narratives, confederates, or actors featuring blameless young children, orphans, or adults in acute pain (e.g., Mehrabian and Epstein, 1972; Batson et al., 1988; Eisenberg et al., 1991). This approach allowed researchers to successfully predict observers' prosocial response from their trait or state empathic concern (EC), personal distress (PD), perspective taking (PT), emotion regulation, and similarity to the target (among other things; see review in Piliavin and Charng, 1990). While this was a highly successful approach to studying observers of distress and need, it did not allow us to understand how real people exhibit need or how their naturally varying responses influence prosocial behavior.

There are significant theoretical reasons to assume that how people display need influences the help they receive. For example, because the willingness to help is known to increase with the salience of the target's need (Dovidio and Gaertner, 1999; Preston, 2013), observers are unlikely to know someone needs help if they do not overtly express distress (Zaki et al., 2008). However, people also withdraw support when they become personally distressed by targets or cannot regulate their own emotional response (e.g., Batson et al., 1983; Eisenberg et al., 1994, 1998)—conditions that increase with the target's level of distress. Thus, individuals in need face a conundrum in which small displays of distress may not make their need salient enough but larger ones may overwhelm observers. Taken together, empathy-based motivational theories of altruism could assume that help is optimally elicited by intermediate levels of distress, but impeded by too little or too much.

Despite this delicate but seemingly logical situation, empathy and emotional resonance can also occur for positive states that can sometimes be even more motivating, fulfilling, and rewarding to observers (Preston and Hofelich, 2012). For example, research on altruism from economics and evolutionary biology that rarely interacts with the empathy-altruism research described above suggests that people should direct resources toward those who can provide substantial return benefits to the giver (Trivers, 1971; Seyfarth and Cheney, 1984; Andreoni, 1990; Noë and Hammerstein, 1994; Brosnan Sarah and de waal Frans, 2002; Fehr et al., 2002, 2005; Gintis et al., 2003; Fehr and Rockenbach, 2004; Preston, 2013). In this framework, clearly distressed targets may actually be passed over in favor of more positive ones when the latter are viewed as offering greater potential return rewards, such as a more enjoyable prosocial interaction, a shared bond, and the feeling that the target's resilience may render them better able to benefit from the aid and to return the help later. Thus, unlike empathy-altruism theories, models that emphasize cooperation and reciprocity (e.g., Trivers, 1971; Gintis et al., 2003) or a cost-benefit analysis (Dovidio et al., 2006) may actually favor positive over distressed targets, especially when their need is similar.

Such complexities are exacerbated by the fact that people have different display rules guiding how emotions should be expressed (Ekman, 1971; Matsumoto, 1990; Zeman and Garber, 1996; Brody, 2000), which in turn influence how much emotionality (particularly negative emotion) they permit in others (Zeman and Garber, 1996; Brody, 2000). For example, people from more stoic cultures may be expected to silently endure the pain of illness, while those from more expressive groups may welcome the opportunity for a “good cry,” while still others may want targets to cover their concerns with jokes or “gallows humor.” Mismatches between the display rules of targets and observers would make it even harder for targets to maximize their potential aid. For example, expressive observers may not realize when a stoic target is in pain while expressive targets may make stoic or suppressive observers feel uncomfortable or judgmental, even if each of those displays would produce a strong response from someone in their own subculture.

Of course, distress is often a typical and honest signal of need that should promote aid in emergency situations, like those studied in the bystander apathy (Darley and Latané, 1968; Latané and Rodin, 1969) or empathy-altruism (Batson, 2011) paradigms. In such situations, positive affect would be incongruous in targets and unlikely to promote aid. Thus, aid in acute cases should be given in proportion to the target's distress or need when the observer can help (see Preston, 2013). However, such acute and immediate need—the focus of most existing research—may not actually be the most frequent form that we encounter in the real world.

Much of our daily altruism is in response to the sustained difficulties of familiar people that we often learn about indirectly during the natural course of conversation. For example, one parent may chat with another at the playground or coffee shop about the stress associated with an illness or pending move, in their own family or that of a common friend. The receiver may subsequently offer support through meals or childcare while further sharing this information with others who may also come to offer help, and so on. These less acute displays have yet to be examined, despite pervading daily life and making the difference between spending one's weekend working or shopping at the mall vs. cooking or babysitting for an ailing or overwhelmed relative or friend.

In sum, there are important reasons to assume that the display of affect during need is a complex problem that is solved in different ways by psychological vs. economic or biological theories. On the one hand, the overt expression of distress or need engenders empathy and altruism, but in a tenuous manner that is easy to under- or overshoot. On the other hand, a positive and resilient response may actually elicit more aid from those seeking to enjoy and build social bonds. Because past research largely aimed to prove the existence of a “pure” form of altruism, and only examined observers, we know little about these potential real-world interactions between targets and observers, which have great practical importance in situations like patient care, parental responding, and cross-cultural interactions.

The first goal of the current study was to document natural variation in the display of need in real-world targets of need that are in a more typical and conversational setting rather than one of acute need. Real hospital patients were used as the targets because illness is a common stressor that people are likely to encounter in relatives, friends, and neighbors who also likely display this need in various ways. Videotaped interviews with patients about their quality of life were used as the stimuli because they displayed real affect and mimicked the more conversant and less acute way that people often learn about need in real life. Hospital patients are also generally regarded as deserving help and differential observer responses to their emotions would have important implications for public health. We hypothesized that there would be variation in the way that the targets presented their need, which could be generalized to include at least (1) a highly distressed type that clearly displayed need and negative affect related to that need, (2) a highly positive type that remained socially engaged and engaging throughout the conversation even when need was clearly present, and (3) a more laconic or reticent type that did not openly express emotion, positive or negative.

The second goal of the current study was to document changes in the way that observers responded to these different natural types of patients, with some main effects observed across observers, such as increased helping for patients with more clear need, and some interaction effects with the observer's own trait propensities. The positive patients were expected to engender high levels of empathy and helping despite not appearing as distressed or in need because they would be more attractive as social partners and would not overwhelm observers the way that highly distressed patients could (e.g., Batson et al., 1983; Eisenberg et al., 1989). Trait empathy was expected to promote giving to the targets who displayed the most clear need (the distressed ones) while PT was expected to promote empathy and helping for targets who were less expressive and do not clearly display their need (the reticent ones) (Preston and de Waal, 2002; Preston and Hofelich, 2012).

Study 1 first measured the response of participants to patient videos that included all visual, sound, and semantic cues, in order to group patients into natural affective types and study the response of observers to each type. To minimize effects of variables other than affect on the prosocial response, videos only included answers to the same four semi-structured questions about quality of life. Information about patients' diagnoses and illness severity was not provided. All research was approved by the Institutional Review Board at the University of Michigan and all participants provided informed written consent before participation.

## Study 1

In Study 1, participant observers (hereafter, “observers”) viewed 14 videos of hospital patients being interviewed about their quality of life (hereafter, “targets”). During each video, continuous measures of observers' heart rate, respiration, skin conductance, and facial muscle activity were recorded. After each video clip, observers self-reported the emotion they perceived in the target [*other*], felt themselves [*self*], and their prosocial response (after Batson et al., 1997). At the end of the study, observers filled out demographic information including trait empathy. A combination of factor and cluster analysis was used to classify the targets into display “types” (hereafter, “target types”) based on their displayed affect in the *other* ratings. Differences in the emotional, psychophysiological, and prosocial response to each target type were examined.

### Materials and methods

#### Targets

The targets were hospital patients with a variety of serious chronic or terminal conditions (cancer, heart disease, Hepatitus C, liver malfunction requiring dialysis). They were videotaped in their hospital room during interviews for an unrelated public health study (e.g., Zickmund et al., 2003). Patients faced the camera while seated or partially reclined with their upper body, face, and head visible along with some surround (e.g., edge of bed, wall behind). Interviews were edited to contain patient responses to the same four questions, which evoked the largest range of affective responses: (1) What has been the impact of your illness on your quality of life? (2) What are your health-related worries? (3) What has been the hardest thing for you to cope with related to your illness? and (4) What in your life are you the most proud of? The questions and their answers were always played in that order, separated by a brief fade. The average clip length was 88 s (range 31–150 s). The specific illness was not mentioned and subjects were unaware of patients' prognoses.

#### Observers

Observers were recruited through advertisements in the daily newsletter of a university hospital and paid for their participation. Fifty-one adults were tested (27 women; mean age = 29.9, range: 19–56), excluding those with a history of neurological or psychiatric illness.

#### Questionnaire data

After each video, observers answered Likert scale questions from 1 [*not at all*] to 7 [*extremely*]. Observers either rated 26 emotion adjectives on how the patient appeared to feel (*other*) and then how they themselves felt (*self*), or vice versa (order counterbalanced across subjects). Adjectives were taken from Batson et al. (1997) including those normally associated with EC (sympathetic, softhearted, warm, compassionate, tender, moved) and PD (alarmed, grieved, troubled, distressed, upset, disturbed, worried, perturbed) as well as adjectives that are traditionally collected but not analyzed (happy, amused, afraid, concerned, disconcerted, horrified, panicked, sorrowful, bothered, pleased, sad, angry). Observers also rated other reactions to the patients on a scale from 1 [*not at all*] to 7 [*extremely*] (except where noted) including “How much do you like the person in this clip?” “How severe do you think this person's illness is?” “How compelled do you feel to help this person?” and “How much help would you offer this person?” [the highest response to this was labeled with the anchor (*as much as possible*)].

After viewing and rating all 14 target videos, observers reported on their gender, age, career, and prior experience with illness. None of these variables had results that were both significant and interesting for the current aims and are not reported here. Participants also completed three trait empathy scales: The Mehrabian and Epstein Scale of Emotional Empathy (ME; Mehrabian and Epstein, 1972), the Interpersonal Reactivity Index (IRI, with subscales for EC, PT, PD, and fantasy (FS); Davis, 1983), and the Jefferson Scale of Physician Empathy (JS, designed to measure empathy for patients; Hojat et al., 2001). All three scales were administered because they tap different aspects of empathy that may be relevant in response to different target types.

#### Psychophysiological data

Psychophysiological variables were averaged across the length of each target video. Mean heart rate [in beats per minute (BPM)] was collected using lead II EKG, with one electrode attached inferior to the costal margin and the other anterior to the sternocleidomastoid muscle. The number of peaks in the skin conductance response (SCR) was measured using electrodes attached to the thenar and hypothenar areas on the palms of both hands and was smoothed and averaged between left and right hands. Facial electromyogram (EMG) responses were recorded with pairs of electrodes attached to the *zygomaticus major* and *corrugator supercilli* muscles and were root-mean-square transformed before averaging. Data were sampled at a rate of 200 Hz using a Biopac MP100WS system (Biopac Systems, Santa Barbara, California) and were analyzed with AcqKnowledge III software for Mac (Biopac Systems). For all measures, the average response across the video was standardized within participant, across the 14 videos, to provide observers' relative response across targets.

### Analysis and results

#### Overview

Patient emotions were first determined through principle components analysis (PCA) of *other* ratings, which were then classified using cluster analysis into target types. Next, we compared the response of observers to each target type, after averaging all targets of a type together (comparing PCA-reduced *self* emotion ratings, psychophysiology, and prosocial responses). Lastly, we attempted to determine if prosocial responses could be predicted from observers' trait empathy. Detailed statistical information for each test is provided with the result below. All tests were evaluated at alpha = 0.05 and *post-hoc* comparisons were Bonferroni-corrected; any comparisons not reported were nonsignificant (*ns, p* > 0.05).

All analyses that included emotion adjective ratings included the order of presentation—*other* or *self* ratings first—because *other* ratings were statistically higher when administered before vs. after *self* ratings (*M* = 2.85, 2.12, respectively), *F*_(1, 49)_ = 15.88, *p* < 0.001, and the effect of order differed by adjective, *F*_(25, 1225)_ > 6.9, *p* < 0.001.

#### Which emotions do targets display? factor analysis of other emotion ratings

**Figure 1:**
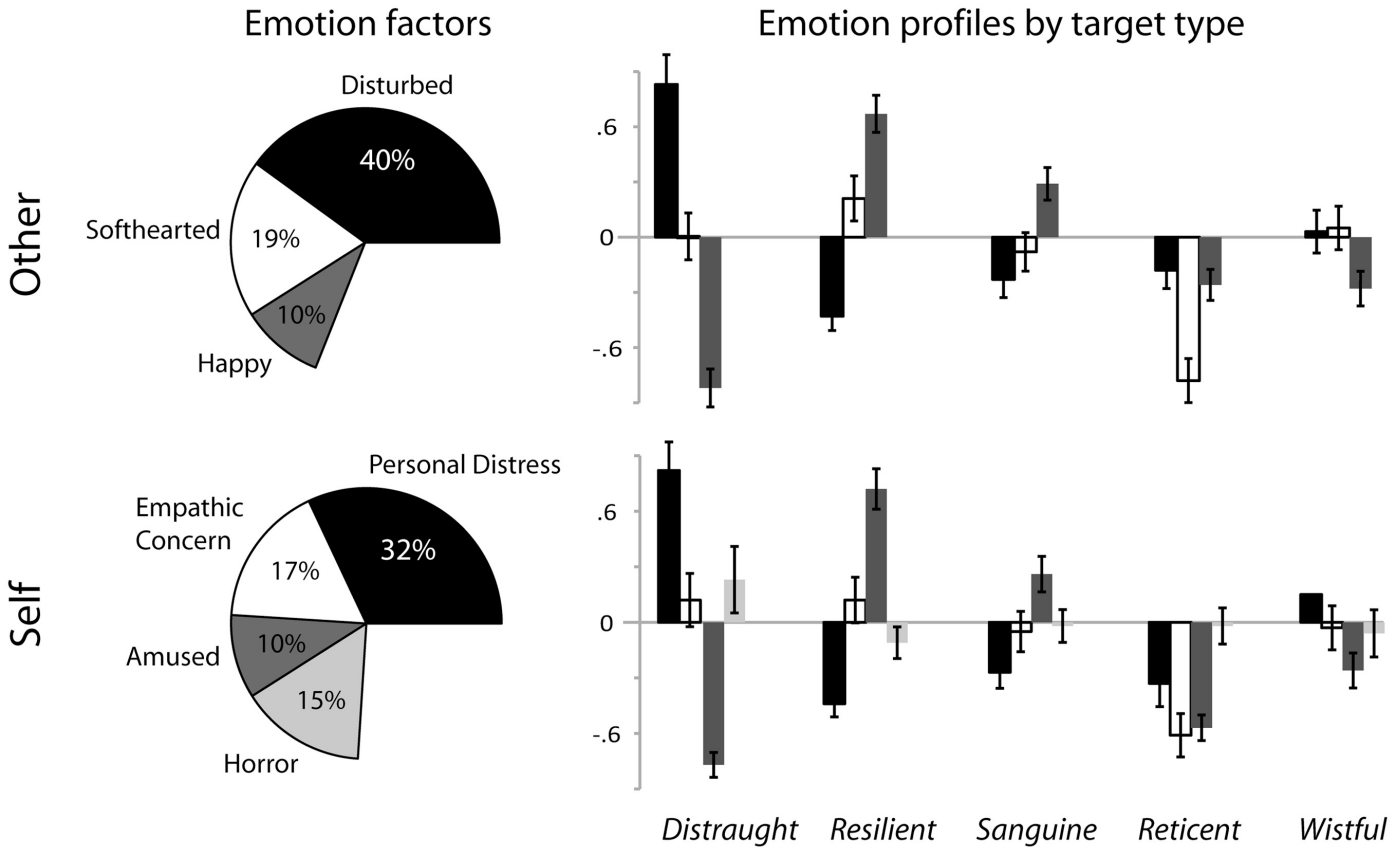
**PCA emotion factors were similar between ratings of the *other* (the targets, represented on the top left) and *self* (the observers response to the targets, represented on the bottom left).** The factor label and percent of variance that it explained is indicated outside of and inside of each pie slice (respectively), with the unexplained variance left out of the pie. Similar factors between *other* and *self* are shaded the same (i.e., disturbed and personal distress are black, softhearted and empathic concern are unfilled, happy and amused are dark gray and horror is light gray). The emotion profiles of the targets **(top left)** and the observers **(bottom left)** are displayed through bar charts representing the degree to which each target type exhibited and elicited each emotion factor (respectively, using means and standard errors of factor loadings averaged across targets within a type). (Horror emerged before amused but is represented last to preserve the similar mappings of emotion factors between targets and observers). Levels of significance are reported in Table 1.

*Other* emotion ratings were standardized within subjects across videos, creating relative differences for each observer across targets that were factor analyzed with PCA. Factors with an eigenvalue >1 were Varimax (orthogonally) rotated. *Other* emotion ratings produced three primary factors explaining 69% of the variance (Figure 1). We report all adjectives that loaded >0.5 on each factor from highest to lowest coefficient, with the adjective bolded if it was used as the factor label. The first, highest adjective was used as the label whenever possible but the third *other* emotion factor uses the third adjective so it can be differentiated from the *self* factor with the same first adjective (below).

The first *other* emotion factor represented the degree to which the target felt “disturbed” (**disturbed**, upset, afraid, bothered, panicked, distressed, disconcerted, troubled, perturbed, worried, sad, horrified, angry, sorrowful, grieved, alarmed, concerned). The second factor represented the degree to which the target appeared “softhearted” (**softhearted**, compassionate, tender, warm, sympathetic, moved). These two factors largely replicate the PD and EC factors found in prior work (respectively, e.g., Batson et al., 1983; Eisenberg et al., 1989; Batson, 2011), but because they refer to qualities of the target and not the observer, those terms are not used here. The third factor represented the degree to which the target appeared “happy” (amused, pleased, **happy**), which is a novel factor that has never been reported in prosocial behavior research using similar methods with fictionalized stimuli.

#### Can the target emotions be used to group them into affective types?

To group the 14 patients by their affective displays, mean *other* emotion factor coefficients were submitted to cluster analysis using the Ward Method (Ward, 1963). The saved PCA coefficients for each extracted *other* factor (above) were averaged across observers per target to create a single mean coefficient per PCA factor, per target type. The resulting profile of emotion factors displayed by each target type was then used to characterize each target type. To statistically characterize them, repeated-measures (RM) ANOVA compared *other* emotion factors within and across target types. The target types were named to best capture their global appearance and the emotions differentiating them, attempting to use terms from the literature whenever possible (esp. *sanguine* and *resilient*).

The clustering technique grouped targets into five types (means and *post-hoc* comparisons in Table 1). From within-type comparisons, the first included three *distraught* targets who were significantly more disturbed than softhearted or happy, and less happy than softhearted, *F*_(2, 98)_ = 46.81, *p* < 0.001. *Distraught* targets often broke into tears while describing their situation and at points had to stop talking to regain their composure. The second target type consisted of four *resilient* targets who were more happy than softhearted and more softhearted than disturbed, *F*_(2, 98)_ = 35.47, *p* < 0.001. *Resilient* targets talked about their struggles, but remained positive and made lighthearted comments or smiled during the interview. The third target type consisted of three *sanguine* targets who were more happy than disturbed or softhearted, *F*_(2, 98)_ = 8.96, *p* < 0.001. *Sanguine* targets were less emotional than *distraught* or *resilient* targets; they talked at length without conveying major health concerns and sometimes made jokes. The fourth target type consisted of one *reticent* male who was less softhearted than disturbed or happy, *F*_(2, 98)_ = 9.92, *p* < 0.001. The *reticent* patient was laconic, giving only the briefest of responses (e.g., single words such as “fine” or “none”), and did not express overt emotion. The fifth and final type consisted of three *wistful* targets who were more disturbed than happy, *F*_(2, 98)_ = 3.73, *p* = 0.03. *Wistful* targets talked quietly about their health problems or fears of dying but did not exhibit overt negativity or distress as *distraught* targets did. The five target types also exhibited differential levels of each emotion factor from one another, as expected from the clustering technique, *F*s_(4, 196)_ > 27.58, *p*s < 0.001 (Figure 1, Table 1). In general, *distraught* targets appeared more disturbed and less happy than all others, *resilient* targets were conversely less disturbed and happier than all others, and the *reticent* target was less softhearted than all others.

**Table 1:** **Mean factor scores, psychophysiological (psychophys.) responses, and ratings by target type in Study 1**.

		**Target types**				
		**1**	**2**	**3**	**4**	**5**
	**Demographic characterization:**	**1FYAC, 2FOAC**	**2FOAC, 2MOAC**	**1FOAC, 1MOAC, 1MOAAA**	**1MOAC**	**1FOAC, 1FYAC, 1MYAC**
		***Distraught***	***Resilient***	***Sanguine***	***Reticent***	***Wistful***
*Other* factor scores	Disturbed	0.83^1a^	−0.43^1b^	−0.23^1c^	−0.18^1cd^	0.03^1d^
	Softhearted	0.004^2ab^	0.21^2a^	−0.08^1b^	−0.78^2c^	0.05^1ab^
	Happy	−0.82^3a^	0.67^3b^	0.29^2c^	−0.26^1d^	−0.28^2d^
*Self* factor scores	Personal distress	0.82^a^	−0.44^b^	−0.27^c^	−0.33^bc^	0.15^d^
	Empathic concern	0.12^a^	0.12^a^	−0.05^a^	−0.61^b^	−0.03^a^
	Horror	0.23^ns^	−0.11^ns^	−0.02^ns^	−0.02^ns^	−0.06^ns^
	Amused	−0.77^a^	0.72^b^	0.26^c^	−0.57^a^	−0.26^d^
Psychophys. responses	Heart rate	−0.03^abc^	0.22^a^	0.09^ab^	−0.60^c^	−0.15^bc^
	SCR peaks	0.59^a^	0.03^b^	−0.19^b^	−0.72^c^	−0.19^b^
	Zygomatic EMG	−0.33^a^	0.45^b^	0.09^c^	−0.22^ac^	−0.30^a^
	Corrugator EMG	0.30^a^	−0.30^b^	0.09^a^	0.01^ab^	−0.01^ab^
	Respiration rate	−0.02^ns^	0.06^ns^	0.05^ns^	−0.10^ns^	−0.11^ns^
Prosocial responses	Liking	3.85^a^	5.16^b^	4.72^c^	3.35^d^	4.49^c^
	Illness severity	4.63^a^	3.97^b^	4.06^b^	3.26^c^	4.92^a^
	Help compelled	4.29^a^	4.11^a^	4.04^a^	3.08^b^	4.19^a^
	Help offered	4.76^a^	4.84^a^	4.79^a^	4.00^b^	4.78^a^

Superscript numbers represent statistical comparisons of emotions within target types (between row comparisons; used for other scores to characterize target types). Subscript letters represent statistical comparisons between target types for each measure (i.e., between column comparisons of other and self emotion factors and prosocial responses across types). Demographic information about the targets in each type are provided under each target type number (F, female; M, male; YA, Young adult; OA, Older adult; C, Caucasian; AA, African American).

#### Which emotions do observers feel in response to targets? factor analysis of observers' self emotion ratings

To examine how observers responded to the five target types, *self* emotion ratings were classified into factors as above. After standardizing the *self* emotion ratings within subjects and across videos, PCA reduced the 26 *self* adjectives into four factors that explained 74% of the variance (Figure 1). Again, factors are presented with adjectives ordered from the highest to lowest coefficient (including any > 0.5), with the adjective bolded when used as the label. The first two emotion factors were again similar to Batson's “PD” (troubled, distressed, worried, upset, afraid, grieved, sad, disturbed, bothered, concerned, sorrowful, alarmed, disconcerted) and “EC” (compassionate, sympathetic, softhearted, tender, warm, moved). In this case, we did use his terms as the factor labels because the *self* factors represent observer affect as in the classic empathy studies making it more parsimonious to use those terms rather than the highest loading adjective. The third novel positive emotion factor again emerged, referred to as “amused” (**amused,** pleased, and happy) along with an additional novel negative emotion factor representing an intense negative, alienating response of observers to targets, referred to as “horrified” (**horrified,** perturbed, angry, panicked). Again, using the same emotion adjectives and a similar rating procedure as in prior studies, we found two completely novel emotional responses of observers through the depiction of naturally-occurring affect.

#### Do observer emotions differ across target types? comparing self emotion factors across target types

Using mixed ANOVA observers' *self* emotion factor responses were compared across target types. As was done for the *other* emotion factors, observer factor loadings were first averaged across targets of the same type, including target type as a repeated measure.

Observers responded with significantly different emotions to target types (means and *post-hoc* comparisons in Table 1, Figure 1, main effects reported here). As predicted, *distraught* targets elicited more PD than any other, *F*_(4, 196)_ = 60.58, *p* < 0.001, and tended to elicit more horror than *wistful* patients, *F*_(4, 196)_ = 2.64, *p* = 0.035. *Resilient* targets elicited more amusement than all other types, *F*_(4, 196)_ = 103.79, *p* < 0.001. The *reticent* target elicited less EC than all other types, *F*_(4, 196)_ = 25.27, *p* < 0.001.

#### Do observers have different psychophysiological responses across target types?

Corroborating the self-reported differences reported above, observers' physiological responses also differed across target types (means and *post-hoc* comparisons in Table 1, main effects below). Observers' sympathetic and heart rate responses differed across types, *F*_(4, 188)_ > 8.70, *p* < 0.001, because *distraught* targets evoked more SCR peak counts than all other types and the *reticent* patient evoked a smaller heart rate response than *resilient* and *sanguine* patients. *Resilient* patients also evoked more positive facial muscle activity than the other types, *zygomatic* EMG, *F*_(4, 188)_ = 14.15, *p* < 0.001, and less negative facial activity than *distraught* and *sanguine* patients, *corrugator* EMG, *F*_(4, 188)_ = 5.09, *p* = 0.001. Respiration rates did not differ, *F*_(4, 188)_ = 0.30, *p* = 0.71.

#### Do observer prosocial responses differ across target types?

Mixed ANOVA compared the remaining observer responses across target types, including how likeable they were, how sick they seemed, how much help they would offer them, and how compelled they felt to help (averaging each observer's response to all targets within a type as above; means and *post-hoc* comparisons in Table 1). All ratings differed significantly across the five target types, *F*s_(4, 196)_ > 18.32, *p*s < 0.001. The *reticent* target was less well liked, seemed less sick, received lower offers of help, and elicited a lower compulsion to help than all other types. After the *reticent* target, *distraught* targets were also significantly less well liked than the remaining three more positive types (*resilient, sanguine, wistful*). *Distraught* and *wistful* targets were also perceived as being sicker than all others.

Although helping differed across target types, observers offered highly similar amounts of help to each type, *r*_(51)_ > 0.84, *p* < 0.0001. To examine relative preferences, two types of frequency data are reported. After removing 9 observers who offered identical amounts of help to all types and 7 who offered their highest amount to more than one (i.e., ties that do not indicate a singular preference), 35 observers gave a higher level of help to one particular type. The greatest number (11) preferred *distraught* targets, but almost as many preferred *resilient* (10) and *wistful* (9) targets and still some preferred *sanguine* (4) and *reticent* (1).

We also compared how often a particular type received more aid than the observer's mean (difference > 0), which includes more data by allowing ties and can be used to intercorrelate preferences. The frequency of preferences over an observer's mean was fairly evenly distributed across target types (*distraught* (29), *wistful* (29), *sanguine* (27), and *resilient* (25) patients), but many fewer observers offered more than their average aid to the *reticent* patient (6). Thus, as with the emotion data, observers did not so much approve of one particular type as much as they failed to empathize with the *reticent* patient. However, observers who preferred some target types gave systematically less to others. Those who offered more help to the calm, *sanguine* patients also offered significantly less to the overtly *distraught* patients [and *vice versa, r*_(51)_ = −0.37, *p* < 0.01] while observers who offered relatively more to the *reticent* patient also offered less to all three positive types [*resilient*: *r*_(51)_ = −0.50, *p* < 0.001; *sanguine*: *r*_(51)_ = −0.59, *p* < 0.0001; *wistful*: *r*_(51)_ = −0.55, *p* < 0.0001]. Thus, observer preferences promote aid for some targets while inhibiting it for others.

#### Is the help observers offer to each target type a function of their trait empathy?

Even though observers offered similar aid across target types, their offers were still associated with trait empathy (detailed statistics provided in Table 3), particularly for *distraught* and *resilient* targets but less so for *wistful* ones. Offers of help increased for *distraught, resilient*, and *reticent* targets across trait empathy measures (i.e., ME, JS, IRI-EC), *r*_(44)_ > 0.30, *p* < 0.05 and increased toward *resilient* and *reticent* patients with PT (IRI-PT), *r*_(44)_ > 0.30, *p* < 0.05. *Sanguine* patients also tended to receive greater offers from those with higher trait empathy or PT (IRI-EC, IRI-PT, JS), *r*_(44)_ > 0.28, *p* < 0.06, while *wistful* targets only received marginally more from those with higher empathy for patients (JS), *r*_(44)_ = 0.28, *p* = 0.06. Both *wistful* and *distraught* targets received marginally less help from observers with higher PD (IRI-PD), *r*_(44)_ < −0.25, *p* < 0.1. These effects were largely replicated with how “compelled to help” observers reported feeling, particularly for trait empathy (ME, JS, IRI-EC; see Table 3).

Observers' trait empathy also predicted preferences to offer more than their average help to specific types (using the difference scores from above). The *reticent* target received relatively more help from those with greater PT, *r*_(44)_ = 0.36, *p* = 0.018, suggesting that active participation in his plight could compensate for his minimal affect. In contrast, *wistful* targets actually received less help from observers with higher PT and EC, *r*_(44)_ < −0.321, *p* < 0.04, suggesting that these subscales may access the response to clear distress that is absent in *wistful* patients. *Resilient* targets were offered more relative help as observers' empathy for patients increased (JS), *r*_(44)_ = 0.33, *p* = 0.03, which measures the extent to which people believe in empathic patient care.

### Discussion

For the first time we measured the affect of real hospital patients to assess how people typically convey need in such serious situations in a fairly natural and conversational setting. These individuals clearly expressed emotion in different ways, but we were also able to group them into a few major types from their displayed emotion, which elicited distinguishable responses in experimental observers.

The targets were classified through their expressed affect into five types: *distraught, resilient, sanguine, reticent*, and *wistful*. Using similar self-report techniques as before (e.g., Eisenberg and Miller, 1987; Eisenberg and Strayer, 1987; Batson et al., 1988, 1997), we discovered that not only do observers normally feel distressed or empathic toward targets, targets also express these emotions, supporting a perception-action (Preston and de Waal, 2002) or emotional contagion (Rapson et al., 1993) view of empathy.

These real targets expressed a surprising amount of positive emotion and elicited very positive feelings in observers—a fact that clearly influenced observers' response even though such feelings are almost never emphasized in typical experiments. Moreover, using real targets of need, we identified another novel emotional response in observers: horror. While the patients in the videos were not currently or acutely experiencing pain, their conversation elicited this very negative response in observers, particularly to the most dysregulated and distressed *distraught* targets. Such a response is understandable, but has gone unrecognized to date even though it would have important implications for support in the real world. For example, horror could predict the withdraw of aid better than PD, since PD actually predicts giving in many cases and is usually intercorrelated with EC. The fact that we revealed two novel emotion factors is particularly striking given that we used similar methods and the same 26 self-report adjectives as in prior work; only the stimuli differed. Moreover, these novel factors—positive emotion and horror—each explained as much variance as EC, suggesting that they are equally important components of the response. Note that the horror factor only emerged in observer *self* ratings and not their *other* ratings of the targets. It is expected that horror could be expressed by targets of need in other contexts, but while discussing one's personal experience with illness it appears more likely to be felt by observers and merged with the other disturbed emotions in the targets.

*Distraught* patients were seen as highly disturbed, distressed, and severely ill and elicited PD, autonomic arousal and negative facial affect in observers. Observers also did not like these patients as much and tended to offer them less help when they were more prone to feel PD. However, their high display and elicitation of PD did not preclude them from receiving help—indeed, these patients actually received the highest offers of help across measures. Thus, people do seem sensitive to need above and beyond the rewards they expect to receive from the target and PD should not be considered as a solely inhibitory response to giving. Of course, the offers of help in this case were hypothetical and did not require interaction with the disliked individuals; moreover, observers could still experience a “warm glow” from helping them if that reward were yoked to the patients' level of need or how difficult it was to help them. Yet, it is striking that almost a third of observers gave the most help to these patients, despite having multiple more likeable ones to choose who had similar illnesses.

That being said, and in support of economic and biological theories of altruism, almost as many observers offered their greatest aid to the *resilient* targets who were perceived as amusing and likeable, elicited positive facial affect, and seemed less sick. *Sanguine* targets were also perceived as happy and amusing, but did not elicit the same positive facial affect, reported liking, or offers of help as *resilient* targets, presumably because they displayed less positivity and need.

Patient preferences also interacted such that observers who preferred to help the calm, *sanguine* patients offered less to overtly *distraught* ones and those who preferred the *reticent* patient offered less to the positive patients. Thus, not only do targets of need differ from one another, and elicit different responses in observers, observers also prioritize certain affective styles and penalize opposing ones, based on the degree to which the targets exhibit overt emotion. These preferences sat atop generally similar offers of help across targets, but even small preferences have real-world consequences as people typically can only help one person at a time while ignoring others. Moreover, despite limited variance, these preferences could also be predicted by observers' trait empathy and PT. In general, more empathic observers offered more help across all types, but particularly toward the emotive *distraught* and *resilient* ones. PT also seemed to help observers identify less salient target need, such as in *resilient* and *reticent* and to some extent *sanguine* targets.

Taken together, real people express their need in a variety of ways, even under highly similar situations, and these differences interact with the affective traits and preferences of observers. Of course, there are limitations. While all patients were hospitalized for serious or life-threatening illness, they had a variety of illnesses at different stages. Thus, the differential responses to the patients could have been influenced by inferences about their illness or what they said and not just their emotion. Notably, even though the *distraught* and *wistful* patients were rated as the most sick and in need, we do not believe they were actually the most sick, using the threat of death as the metric of severity. Multiple patients in the more positive *resilient* and *sanguine* target types had much more life threatening illnesses than the *distraught* ones. One sanguine patient died in the same week as the interview despite not even displaying enough need to be classified as *resilient*. However, there could be lawful relationships between the type and severity of people's illness and their affect. For example some cancer patients who are regarded as resilient also engage actively in meaning making processes (Park, 2010), which may be more pronounced in those close to death. However, the prosocial response of other people to them is expected to be more powerfully driven by their expressed affect over and above their need state. To demonstrate the power of affect alone, apart from any cues about their illness or situation imparted during the interviews, a second study was performed.

Study 2 showed new observers the same videos, but with the semantic content stripped through an audio filter that made the words too garbled to understand while preserving the emotional prosody and facial affect. The observers also rated each patient's apparent health status to use as a covariate. This way, any replication of the emotion factors and patient classification without sound and taking apparent health into account could be directly attributed to their expressed affect. In addition, to address unrelated concerns that *self* and *other* ratings in Study 1 influenced one another (e.g., subjects giving the same rating for both, anchored to the one they did first), observers in Study 2 only rated one or the other. Finally, real monetary donations were added to determine whether offers of support would be similar when the offer was not hypothetical. A rank-ordering of patients was also added in case offers did not vary strongly across types. Most of the results from Study 1 were expected to replicate, but fine-grained distinctions among the targets were expected to be lost in the total absence of semantic cues.

## Study 2

### Introduction

Study 2 aimed to verify that (1) similar emotion factors and target types would emerge when only visual and affective cues were available (without verbal content and when people only rate patients' or their own emotion), (2) observers would have similar affective and prosocial responses to the targets under these conditions, (3) the results would hold after controlling for perceived patient health, and (4) offers would show similar patterns when observers had to donate real money. To increase the sample size for statistical power, Study 2 was conducted online so psychophysiological data were not collected.

### Materials and methods

#### Targets

The same 14 patient videos from Study 1 were used in Study 2 but the sound channels were modified to render the spoken words unintelligible. Sound was removed from the portions of the interview where the interviewer spoke offscreen and his questions were printed on the screen so participants could understand the context of patients' responses. Audio from the patients' responses was then altered with a band pass filter between 102 and 750 Hz and a +9.5 dB band at 270 Hz (*Q* = 1.0); this eliminated high frequency sounds while preserving emotional prosody and tone of voice. Participants were explicitly told that the sound had been altered to be difficult to understand because we were interested in their perception of and response to patient emotion, above and beyond their speech content. As a manipulation check, all participants rated how much verbal content they understood after responding to each video (1, *nothing*; 2, *one or two words*; 3, *a few words here and there*; 4, *a few partial sentences*; 5, *most of the content*; 6, *all of the content*).

#### Observers

Ninety-nine adult participants were recruited from Amazon Mechanical Turk (aka, “Mturk”; https://www.mturk.com/mturk/welcome) to watch and rate the videos. Forty-nine participants rated only the emotions of the patients (*other*; 32 women; mean age: 37.1, range: 18–74) and fifty different participants rated only their emotional response to the patients (*self*; 35 women; mean age: 33.56, range: 18–59). Participants were compensated $0.75 for participation, plus any money they chose not to donate (described below).

#### Perception of targets

After each video, participants in the *other* condition rated the targets on their displayed emotion through the same 26 adjectives as in Study 1. They also rated them on aspects related to the patient's perceived health: how sick the patient seemed, how energetic, their apparent prognosis from recovering to dying, how much emotional support they needed (“e.g., talking to them, giving advice, soothing, spending time with them”) and how much practical support they needed (“e.g., getting prescriptions, changing sheets, watering plants, grocery shopping”).

#### Observer response to targets

Participants in the *self* condition rated their emotional response to each patient using the same 26 adjectives as Study 1 as well as how much emotional and practical support they were willing to give each patient, and how much they liked them. After these ratings, participants were told that the patients were interviewed in exchange for monetary donations to help with their illness and to promote awareness for their disease. They were allotted five tokens per patient and told that they could donate any number of them to the patient. They were explicitly told that any tokens they did not donate would be converted to cash at the end of the study and paid to them as a bonus in Mturk. The token exchange rate was intentionally not provided because research in our lab found that participants who perceive the total amount to be low give all tokens, precluding the variance necessary for analysis. In the event that observers again gave highly similar amounts across all patients, we added a ranking task after all videos in which participants drag-and-dropped thumbnail images of patients into order from the one they most-to-least wanted to help (1–14, respectively). To focus on the relationship between observers' emotional and prosocial response, only ranking data from observers in the *self* condition were analysed.

#### Trait scales

At the end of the study, participants completed the IRI as in Study 1 to assess individual differences in trait empathy and completed the Berkley Expressivity Questionnaire (BEQ; Gross et al., 2000) to determine if differences in expressivity could predict target preferences.

### Analysis and results

Confirming that the sound was successfully altered, participants reported only understanding one or two words across all videos (*other*: *M* = 2.39, *SD* = 1.21; *self*: *M* = 2.21, *SD* = 1.25). Next we determined if the patients were perceived and responded to similarly in this condition.

#### Do similar patient types emerge when verbal content is eliminated?

Analysis was as in Study 1, with PCA factor analysis reducing *other* emotion ratings into factors, which were clustered with the Ward method into target types. Three target emotion factors again emerged, replicating those in Study 1 and explaining 57% of the variance (listed with all adjectives with >0.5 loadings from highest to lowest coefficient). The first was the “disturbed” factor (panicked, horrified, upset, afraid, distressed, worried, bothered, sorrowful, sad, grieved, perturbed, concerned, troubled, alarmed, disconcerted, angry). The second factor had only strong negative loadings indicating feeling *not amused*, capturing the “happy” factor from Study 1 (amused, funny, pleased, happy). The third factor replicated the “softhearted” factor (softhearted, tender, compassionate, warm, sympathetic, engaging, likable).

As in Study 1, a five-cluster solution was extracted and similar target types emerged, particularly the distinction between lacking affect, high distress, and positive affect. The *reticent* patient again separated from the rest; the *distraught* patients again clustered together (although now split across two clusters); and one large cluster combined the positive patient types into one group (*resilient, sanguine, wistful*). One *resilient* and one *sanguine* target formed a new cluster.

#### Do emotional responses to the original patient types vary when content is eliminated?

To determine if observer responses to patient types remained after verbal content was eliminated and observers only rated their own emotion, PCA factor analysis reduced the 26 *self* emotion adjectives into factors, which were averaged across targets in the five original types. Observer responses were modeled by averaging responses into the five clusters from Study 1 because the goal was to determine if observer responses to these original types would replicate when observers only had access to their affect. Based on the scree plot, four factors best explained observer *self* emotions, accounting for 45.4% of the variance. The first factor combined the “PD” and “EC” factors from Study 1 (PD/EC: sad, sorrowful, worried, concerned, sympathetic, moved, upset, softhearted, bothered, troubled, grieved, distressed, compassionate, tender). The second factor was similar to the “horrified” factor from Study 1 (perturbed, panicked, horrified, afraid, angry), and the third and fourth factors divided the positive emotional response into two factors: “happy” (warm, likable, happy) and “amused” (funny, amused).

RM-ANOVA compared observer responses (*self* emotion factors) within and across the five original target types, which again differed significantly (means and *post-hoc* comparisons in Table 2). The five original types still elicited significantly different PD/EC and horror in observers, *F*s_(4, 188)_ > 6.51, *p* < 0.001, as *distraught* targets elicited more PD/EC than any other type and elicited more horror than *reticent* and *wistful* targets. *Resilient* targets also made observers feel more happy than *distraught* patients, *F*_(4, 188)_ = 3.91, *p* = 0.004, and more amused than *wistful* patients, *F*_(4, 188)_ = 2.48, *p* = 0.045.

**Table 2:** **Mean emotion factor scores and ratings by target display type (Study 2)**.

		**Target types**				
		**1**	**2**	**3**	**4**	**5**
		**Distraught**	**Resilient**	**Sanguine**	**Reticent**	**Wistful**
*Self* factor scores	PD/EC	0.56^a^	−0.18^b^	−0.04^b^	−0.20^b^	−0.23^b^
	Horrified	0.38^a^	−0.02^ab^	−0.12^b^	−0.01^ab^	−0.23^b^
	Happy	−0.25^a^	0.26^b^	−0.04^ab^	−0.19^ab^	0.002^ab^
	Amused	−0.11^ab^	0.23^a^	−0.10^ab^	−0.06^ab^	−0.07^b^
Prosocial responses	Liking	4.79^a^	5.13^b^	4.90^a^	4.67^a^	4.94^ab^
	Emotional support	5.12^a^	5.04^a^	5.01^a^	4.58^b^	4.92^a^
	Practical support	5.07^ab^	5.04^a^	4.91^ab^	4.60^c^	4.81^bc^
	Ordinal ranking	6.10^b^	6.85^b^	7.23^ac^	8.32^bc^	9.03^a^
	Token donation	3.70^ns^	3.54^ns^	3.51^ns^	3.48^ns^	3.54^ns^

Subscript letters represent statistical comparisons between the display types for each emotion (between column comparisons).

To determine if these effects actually reflect target emotions rather than perceived health status, additional analyses replicated these results after controlling for perceived health. A health composite index was derived from ratings by participants who only rated the targets (the *other* condition) averaging the apparent sickness, energy level, and prognosis within each target and then across all targets in a type to create a single health status index per type. This health status composite was then entered as a covariate into a linear mixed model comparing observers' emotional responses (*self* emotion factors) to the types, nested within observer, with observer as a random factor. All effects remained significant, *F*s_(4, 188)_ > 2.97, *p*s < 0.021.

#### Prosocial self-reported responses

Observer ratings of how much they liked the patient, wanted to give them emotional, practical, and monetary support, and their ordinal ranking were averaged for all targets in the five original types and compared with RM-ANOVA (means and *post-hoc* comparisons in Table 2). Again, *resilient* patients were liked more than all others (except for *wistful*), *F*_(4, 188)_ = 2.90, *p* = 0.02 and the *reticent* patient received less emotional and practical support than any other, *F*s_(4, 188)_ > 3.61, *p*s < 0.007. These effects were still significant after controlling for targets' perceived health status in the linear mixed model, *F*s_(4, 188)_ > 2.90, *p* < 0.02. The order in which observers wanted to assist the target types also differed significantly, *F*_(4, 188)_ = 10.12, *p* < 0.001, with *distraught, resilient*, and *reticent* patients being ranked higher than *wistful* and *sanguine*. These rankings were also predictable from observers' trait data (Table 3) as the relatively calm *sanguine* targets were ranked higher by observers with greater PT (IRI-PT), *r*_(46)_ = −0.38, *p* = 0.007 and lower by those who overtly display more negative emotion in life (negative expressivity subscale of BEQ; rank close to 14 out of 14), *r*_(46)_ = 0.34, *p* = 0.02. There were no other significant relationships, *r*s_(46)_ < 0.22, *p*s > 0.13.

**Table 3:** **Prosocial responses correlated with trait empathy across studies and measures**.

	**Target types**				
	**1**	**2**	**3**	**4**	**5**
	***Distraught***	***Resilient***	***Sanguine***	***Reticent***	***Wistful***
**STUDY 1: HELP OFFERED**					
ME	0.24	0.30^*^	0.22	0.22	0.18
IRI-EC	0.37^*^	0.38^*^	0.29^~^	0.31	0.24
IRI-PT	0.29^~^	0.30^*^	0.30^~^	0.39^**^	0.18
IRI-PD	−0.25^~^	−0.22	−0.17	−0.18	−0.27^~^
IRI-FS	0.10	0.17	0.12	0.08	0.04
JS	0.32^*^	0.40^**^	0.29^~^	0.29^~^	0.28^~^
**STUDY 1: COMPELLED TO HELP**					
ME	0.36^*^	0.47^**^	0.33^*^	0.25	0.27^~^
IRI-EC	0.40^**^	0.41^**^	0.26^~^	0.29^~^	0.25
IRI-PT	0.18	0.21	0.17	0.22	0.10
IRI-PD	−0.08	0.02	0.03	0.00	−0.12
IRI-FS	0.11	0.18	0.22	−0.05	0.04
JS	0.30^~^	0.35^*^	0.35^~^	0.28^~^	0.25^~^
**STUDY 2: ORDINAL HELP RANKING**					
IRI-EC	−0.39^**^	0.19	0.03	0.12	0.12
IRI-PT	−0.09	0.22	−0.38^**^	0.17	0.11
IRI-PD	−0.19	0.16	0.07	−0.11	0.05
IRI-FS	−0.19	0.21	0.04	0.15	−0.11
Pos. Expr.	−0.19	0.12	0.16	−0.13	0.02
Neg. Expr	−0.26^~^	−0.04	0.34^*^	−0.08	0.06
Impulse Str.	−0.33^*^	0.13	0.18	−0.04	0.09
**STUDY 2: TOKENS DONATED**					
IRI-EC	0.16	−0.07	−0.08	−0.05	0.03
IRI-PT	0.06	−0.12	0.16	−0.10	0.02
IRI-PD	−0.12	0.14	0.04	−0.04	0.04
IRI-FS	−0.09	0.14	0.09	−0.04	−0.05
Pos Expr.	0.18	−0.15	−0.34^*^	0.06	0.16
Neg Expr.	0.07	−0.10	−0.18	0.07	0.09
Impulse Str.	0.09	−0.08	−0.22^~^	0.12	0.00

Study 1 used self-reported help (“how much help would you offer”; “how compelled do you feel to help”). Study 2 used different measures to more precisely estimate target preferences and avoiding intercorrelated gifts, including an ordinal target ranking and real monetary donations (using the difference from each observer's mean offer). All measures were first averaged across targets within a type per observer. ME, Mehrabian and Epstein scale of emotional empathy; IRI, Interpersonal Reactivity Index with subscales for Empathic Concern (EC), Perspective Taking (PT), Personal Distress (PD) and Fantasy (FS); JS, Jefferson Scale of empathy for patients; Pos. Exp., Neg. Expr., and Impulse Str. Refer to the positive and negative expressivity and impulse strength subscales of the Berkeley Expressivity Questionnaire (BEQ, respectively). Significance level noted as follows:

** p < 0.01,

* p < 0.05,

~ p < 0.1.

#### Monetary donations

Nearly half of observers (20 of 48) gave the same number of tokens to all patients. The most common offer was giving all tokens and the next most common was giving zero tokens, which precluded significant overall differences across types, *F*_(4, 188)_ = 1.08, *p* = 0.37. Key differences could still be replicated using the frequency analyses from Study 1. Of the 23 observers who exhibited a singular preference (gave more to one group), the greatest number again preferred *distraught* targets (10); the remaining observers had preferences that were evenly spread across remaining types (3 preferred *resilient*, 2 *sanguine*, 4 *reticent*, and 4 *wistful*). Comparing how often observers gave more than their mean amount to a target type, the greatest frequency again preferred *distraught* targets (16), but almost as many preferred *resilient* (14) with a fairly even distribution across the remaining three (9 *wistful*, 8 *sanguine*, 9 *reticent*). We also replicated the intercorrelated preferences across target types from Study 1, as observers who donated more money to *distraught* patients again gave less to *sanguine* patients, *r*_(46)_ = 0.53, *p* < 0.001, and those who gave more to the *reticent* patient again gave less to the three positive types, *resilient*: *r*_(46)_ = 0.50, *p* < 0.001; *wistful*: *r*_(46)_ = 0.40, *p* = 0.005; *sanguine*: *r*_(46)_ = 0.20, *p* = 0.17. *Sanguine* patients received more help to the extent that observers reported not expressing positive emotion in their own life (positive expressivity subscale of BEQ; Table 3), *r*_(48)_ = −0.3399, *p* = 0.0181. Further affirming the validity of the self reported offers of help, emotional and practical support were significantly correlated with the number of tokens donated over each observer's mean, *r*_(240)_ > 0.19, *p* < 0.002.

### Discussion

Study 2 attempted to replicate the results from Study 1 even after eliminating all spoken words and controlling for how sick the patient seemed and requiring offers of real money.

As expected, some fine gradations between target types were lost without the semantic cues (e.g., differences between the *resilient, sanguine*, and *wistful* patients), but Study 1 was largely replicated, particularly the distinctions among high negative affect (two *distraught* types), high positive affect (one large type that combined *resilient, sanguine, wistful* patients), and a lack of affect (*reticent*). Of course, in the real world, our ability to discriminate people employs both verbal and bodily affect, but the effects from Study 1 were surprisingly robust to the perturbations in Study 2.

Importantly, observers had similar reactions to the targets in Study 2. Similar emotional responses (from the *self* factors) emerged in the observers, and our novel emotion factors were even more salient, as the traditional PD and EC combined into a single factor while the positive emotion factor divided into two distinct factors. Without the semantic information, observers again offered less help to the *reticent* patient while liking the *resilient* patients the most. Observers also showed similar target preferences, with more people preferring to help the most needy but disliked *distraught* patients but almost as many preferring the *resilient* patients and some preferring the other types. It is remarkable that people can exhibit such similar patterns of disliking the *reticent* patient, offering the most aid to the less well-liked *distraught* patients, and liking and offering almost equal levels of help to the *resilient* patients, even with the sound so distorted.

The *sanguine* patients across studies elicited particularly interesting interactions with observers' trait tendencies. In Study 1 they tended to be helped more by those with greater PT and in Study 2 they were ranked higher by those with higher PT and lower by those who express a lot of negative emotion. *Sanguine* patients also received relatively larger monetary donations from those who express less positive emotion. Thus, the need of these calm and collected patients may have been too subtle for those who associate need with distress, but was perhaps ascertained by those who carefully attended to them or valued their understatement.

These differences among target types were upheld even after controlling for how sick the patients seemed on multiple dimensions. Moreover, we can anecdotally attest to the lack of connection between how sick patients actually were and how sick they seemed since, for example, a *sanguine* patient died shortly after the videos were taken and multiple *resilient* and *wistful* patients had life-threatening diseases while multiple *distraught* patients had chronic but treatable illnesses. Future research can further examine these relationships in the event that chronic illness is lawfully associated with high negative affect or terminal illness with more detached and sagacious sentiments.

The data generally support an interactionist view of social behavior (Griffiths and Scarantino, 2009; Van Kleef, 2009; Preston and Hofelich, 2012), in which it is not just the observer or the target who dictate the prosocial response, but rather their interaction. For example, emotion-regulation skills influence observers' response to need (e.g., Eisenberg and Fabes, 1992; Eisenberg et al., 1994, 1998) and, thus, those with lower regulatory skills may be more likely to avoid *distraught* patients, even when they have more personal experience with the state. In addition, people from less expressive cultures could punish or avoid *distraught* targets more than those who believe negativity is natural and common. As support, our observers with high trait PD tended to offer less to sad *wistful* and *distraught* patients while people who display a lot of negative emotion were less inclined to help calm, *sanguine* patients, and people who don't display positive emotion were more inclined to help them. *Distraught* patients also evoked the most variable responses; those who preferred the positive *resilient* or *sanguine* targets simultaneously gave less to *distraught* patients. These preferences may reflect observer expectations about how people are expected to react to illness or strife, which could serve as a rich source of data on interpersonal and cross-cultural differences (Preston and Hofelich, 2012). People may also have more intuitive vs. rational or practical decision styles that influence their relative aid across types. For example, *distraught* patients should receive the most aid if observers emphasize need in a simple way while *resilient* patients should be preferred if deciders consider both absolute need and the potential for change, as *resilient* patients may be better able to build upon support to help themselves. These hypotheses are in keeping with cost-benefit views of altruism (Dovidio et al., 2006) but require additional research that offers a rich source of ideas for future work.

## Final discussion

In daily life we are surrounded by people who could use our help. Everyone has needs that would benefit from some help, most of which are not immediate, but many of which are equally or more serious and problematic than the electric shocks or ice buckets of water that are often used in experiments. The neighbor next door has a baby that cries most of the night, an unmarried uncle suffers from cancer and has no one to take care of him, the school needs someone to organize a fundraiser, and a spouse needs help practicing for a job interview. All of these are concurrent requests for our resources—material or nonmaterial—and people must make routine decisions to help only some of them. What predicts these choices?

Most research in psychology has focused on the emotional correlates of helpful observers while examining only a few target qualities like need salience, culpability, similarity, relatedness, age and vulnerability (see reviews in Piliavin and Charng, 1990; Preston and de Waal, 2002; Batson, 2011; Preston, 2013). However, people also vary a lot in how they express need, even in the same situation—variance that influences who wants to help them and how much. The goal of this study was to examine this natural variation and how it affects and interacts with observers and their own preferences.

With a relatively small sample of fourteen real hospital patients, suffering from a variety of serious chronic and terminal illness, we were able to detect at least five subtypes of displayed affect during a time of need: *distraught, resilient, wistful, sanguine, and reticent*. The main affective differences across targets were even replicated in the absence of spoken text, again identifying targets who express a lot of negative affect (*distraught*), a lot of positive emotion infused with some discussion of their plight (*resilient, wistful, sanguine*), and a lack of emotion or desire to discuss personal problems (*reticent*). Our typology is likely not exhaustive, and a sample that is larger or taken from another need context will surely find additional types. However, the complexity of the emotions represented by even just a handful of patients attests to the degree to which people's response to need varies and affects observers in predictable ways.

While observers agreed that *distraught* patients needed the most help, they were also disliked by most observers and even elicited a novel and negative state of feeling horrified, perturbed, angry, and panicked. On the one hand, these results accord with theories that predict the greatest aid for the most salient need (e.g., see Dovidio and Gaertner, 1999; Zaki et al., 2008; Preston, 2013). However, they clearly indicate that high levels of observed distress in targets and PD in observers does not preclude giving (Preston and Hofelich, 2012).

Our results also support economic and biological views that emphasize altruism as a collaborative force in group life (Seyfarth and Cheney, 1984; Noë and Hammerstein, 1994; Brosnan Sarah and de waal Frans, 2002; Fehr and Rockenbach, 2004). Almost as many observers preferred to help the more positive *resilient* patients over the ones in the most need because they still have some need but were better liked. Moreover, nontrivial numbers of observers even preferred the three remaining types even though they were not the most in need or the best liked, including fairly even preference distributions over *sanguine, reticent*, or *wistful* patients, oftentimes predictable by their PT skills. The differential response to *distraught* vs. *resilient* patients provides a particularly promising way to examine observer-target interactions since both have serious need and elicit aid, but the former displays largely negative affect and the latter largely positive. The patient videos and transcripts will be shared with other researchers, and variables that had important effects in this context can be extended to more controlled settings, to further our understanding of these interactionist effects.

Taken together, the light of scientific investigation has been shown for decades upon the traits and emotions of the people who observe need, leaving information about how people express need largely in the dark. By studying prosocial behavior in the context of a naturally-occurring social interaction, which reflects both the quality of the target and observer, we can better illuminate human giving as it occurs in everyday life.

### Conflict of interest statement

The authors declare that the research was conducted in the absence of any commercial or financial relationships that could be construed as a potential conflict of interest.
